# Laser Surface Modification of Powder Metallurgy-Processed Ti-Graphite Composite Which Can Enhance Cells’ Osteo-Differentiation

**DOI:** 10.3390/ma14206067

**Published:** 2021-10-14

**Authors:** Peter Šugár, Barbora Ludrovcová, Marie Hubálek Kalbáčová, Jana Šugárová, Martin Sahul, Jaroslav Kováčik

**Affiliations:** 1 Institute of Production Technologies, Faculty of Materials Science and Technology, Slovak University of Technology, J. Bottu 25, 917 24 Trnava, Slovakia; barbora.ludrovcova@stuba.sk (B.L.); jana.sugarova@stuba.sk (J.Š.); 2 Institute of Pathological Physiology, 1st Faculty of Medicine, Charles University in Prague, U Nemocnice 5, Praha 2, 128 53 Prague, Czech Republic; 3 Institute of Materials Science, Faculty of Materials Science and Technology, Slovak University of Technology, J. Bottu 25, 917 24 Trnava, Slovakia; martin.sahul@stuba.sk; 4 Institute of Materials and Machine Mechanics, Slovak Academy of Sciences, Dúbravská cesta 9, 845 13 Bratislava, Slovakia; Jaroslav.Kovacik@savba.sk

**Keywords:** graphite–titanium composite, laser micromachining, surface morphology, biocompatibility, osteo-differentiation, stem cell

## Abstract

The paper examines the surface functionalization of a new type of Ti-graphite composite, a dental biomaterial prepared by vacuum low-temperature extrusion of hydrogenated-dehydrogenated titanium powder mixed with graphite flakes. Two experimental surfaces were prepared by laser micromachining applying different levels of incident energy of the fiber nanosecond laser working at 1064 nm wavelength. The surface integrity of the machined surfaces was evaluated, including surface roughness parameters measurement by contact profilometry and confocal laser scanning microscopy. The chemical and phase composition were comprehensively evaluated by scanning electron microscopy, energy-dispersive X-ray spectroscopy and X-ray diffraction analyses. Finally, the in vitro tests using human mesenchymal stem cells were conducted to compare the influence of the laser processing parameters used on the cell’s cultivation and osteo-differentiation. The bioactivity results confirmed that the surface profile with positive kurtosis, platykurtic distribution curve and higher value of peaks spacing exhibited better bioactivity compared to the surface profile with negative kurtosis coefficient, leptokurtic distribution curve and lower peaks spacing.

## 1. Introduction

Biomaterials contribute to the life quality improvement of many patients by replacing damaged tissues. The harmed hard tissues are generally replaced by ceramic or metallic biomaterials. Titanium and its alloys, stainless steel and cobalt-chromium alloys are typical representatives of metallic biocompatible materials [1]. The material properties essential for medical purposes include biocompatibility, specific strength, corrosion resistance, high mechanical resistance, low modulus of elasticity and stability [2,3,4]. The biocompatibility of titanium and its alloys is associated with the ability to interact with human tissue without adverse effects of the low ion and particle release into the organism [3,5,6].

The formation of a direct bond between the implant and the bone, called osseointegration, is a series of complex events that are triggered after the implant is inserted into the body [7]. Osseointegration begins with wetting the implant surface, continues with the protein absorption, and is followed by the cell’s adhesion, proliferation and differentiation. The initial state of osseointegration can affect the long-term success of implantation [4]. The final stage is related to the formation of a new bone by the mineralization of the bone matrix on the bone-implant interface by osteoblasts [8,9]. The course of osseointegration is influenced by the properties and reactions of the host tissue on the one hand, and, on the other hand, by the properties of the implant material. In addition to the mechanical properties of the implant material, the surface properties are also important [4]. The properties of implant surfaces that can positively influence the osseointegration process have been investigated in surface engineering with the greatest emphasis on surface wettability, topography, roughness and chemical composition [7,8,9,10,11]. Thus, through surface engineering, the bio-functionalities of implants, such as cells adhesion and growth, as well as tissue regeneration, can be enhanced [5,8].

The surface roughness at the micrometer scale should enhance cell attachment, and differentiation, and the roughness at the nanometer scale increases surface energy [12,13,14]. The roughness, Ra, of the currently applied implants ranges between 1 and 2 µm [9]. From the topography point of view, a surface with grooves or depressions of the same size as the cells (a specific type of cell) is more preferred for its colonization by these particular cells [7]. This phenomenon can be applied in the modulation of the surface to attract the particular cell type, e.g., the preferable attraction of osteoblasts instead of bacteria [15] or gingival fibroblasts instead of oral bacterial strains [16], fibroblast versus osteoblast colonization [17] and many others.

Surface properties leading to improved osseointegration conditions are achieved through advanced manufacturing technologies. These can be divided into additive or subtractive surface modification processes or can be grouped as chemical and physical surface modification methods [12]. The additive surface treatments comprise biochemical surface modifications when the organic molecules are incorporated onto the surface [11]. It includes plasma spraying [18,19], physical or chemical vapor deposition [20,21], different types of coatings, e.g., bioactive [22,23], antibacterial [24], bio-mimetic [25] and nanostructured coatings [26]. The subtractive technologies include machining or micromachining [27], grit-blasting [28], acid etching [29], electrochemical machining [30] and laser treatments [31]. When combining several methods at the same time, the advantages of each of them are fully utilized. The grit-blasting followed by acid etching is the most common combination of surface treatment methods [32,33].

Laser machining is a non-contact technology where the kinetic energy of emitted photons is converted into thermal energy when interacting with a workpiece surface, causing the melting and removal of material from the surface by vaporization, chipping or other erosive processes [34]. Machined surfaces consist of remelted material, which is formed into depressions and protrusions due to the pressure of vapors generated during the evaporation of the material in the place of interaction with the laser beam. It is possible to make functional features, regular textures, laser-induced periodic surface structures (LIPSS), nanostructures, etc. [8]. The advantages are fast machining process, precision, accuracy, versatility and non-contamination of the workpiece [12]. Certain roughness minimization of the heat-affected area and elimination of the cracks on the surface can be achieved by setting the input process parameters in a controlled way [35]. As a technology that provides a suitable alternative to other surface treatment methods of biomaterials, thereby leading to osseointegration improvement, the laser treatment of implants’ surfaces is an object of interest for many researchers around the world [34,36,37]. Borcherding et al. [38] treated a Ti6Al4V alloy with a TiO_2_ coating by laser. They found improved osteoblast adhesion and viability on a surface treated as such. Dumas et al. [39] produced the biomimetic micro and nano textures on the Ti6Al4V surface. They compared mesenchymal stem cells growth and differentiation on textured and polished surfaces. The speed of the cells spreading on the textured surfaces was higher. Moreover, micro-pits with nano-ripples improved the osteogenic potential. Yoruç et al. [40] also focused on the laser treatment of a Ti6Al4V alloy and its subsequent immersion in simulated body fluid. The laser treatment of the surfaces significantly increased the hydroxyapatite precipitation, which was due to the increasing surface roughness contributing to the enhanced osseointegration. Kuczyńska-Zemła et al. [41] used the direct laser interference lithography on the acid-etched surfaces of the commercially pure (cp) titanium grade 2 to form uniform Ca-P coatings on them. They found that osteoblasts preferred to grow along these obtained patterns. Wedemeyer et al. [42] formed nanostructures by laser nanostructuring on the Ti6Al4V alloy coated with titanium niobium nitride and titanium plasma spray. The treated samples were implanted into rabbit femora, and all implants were found osseointegrated and well anchored in the bone. However, the nanostructures had no further influence on the fixation of the implants. A negative effect of the surface structure on osteoblast was described by Babuska et al. [43], who investigated the proliferation of osteoblasts on the laser-treated surfaces of cp Ti grade 2 and grade 4 of different microstructures. They observed that materials with lower average grain sizes exhibited significantly higher wettability. Despite the increased roughness after laser treatment, the proliferation of osteoblasts was worse when compared to the surface without laser treatment.

The influence of laser energy on the machined surface morphology, roughness, and chemistry of the Ti-graphite composite samples, prepared by pioneering a low-temperature powder metallurgy technique, was investigated and evaluated recently [44,45]. The authors illustrated that the amount of thermal energy incorporated in the working material had a remarkable effect on the machined surface and identified the surface profiles that could promote the osseointegration properties. In order to obtain a more complex picture of the human mesenchymal stem cells bioactivity on these surfaces, extended research was carried out using a number of analytical techniques. The research brought new findings that more precisely determined which surface profile parameters of the laser-machined surface have the most impact on the cell’s cultivation and osteo-differentiation. The research results are described in the current paper.

## 2. Materials and Methods

### 2.1. Experimental Material

**Figure 1:**
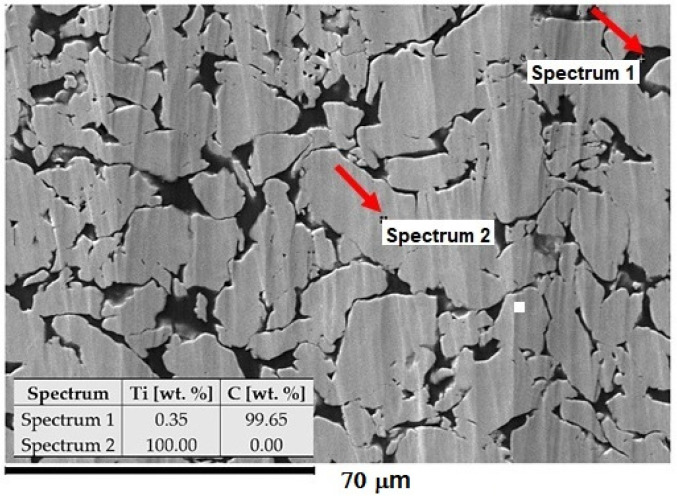
Structure of Ti-graphite composite with the composition of elements. The SEM image was taken at 800× magnification.

In the research, the Ti-graphite composite prepared by the low-temperature powder metallurgy method was used as an experimental material. It consisted of the CP HDH (hydrogenated-dehydrogenated) titanium powder particles with an average size below 32 μm in diameter (Kimet Special Metal Precision Casting Co., Ltd., Hengshui, China), reinforced with 15 vol. % of graphite flakes with an average particle size of 16 μm with a flake aspect ratio of 0.1 and a purity of 99.9%. The wet dispersion-based Fritsch Analysette 22 (FRITSCH GmbH—Milling and Sizing, Weimar, Germany) device for the range of powder sizes 0.5–1500 μm was utilized to determine the powder size distribution. The measured values for titanium powder and graphite flakes were as follows: d_50_ = 24.9 and 5.6 μm and d_90_ = 46.3 and 13.9 μm, respectively. First, the titanium powder with the graphite flakes was dry mixed in a Turbula mixture for 30 min. In this case, a low-temperature powder metallurgy method was used for powder mixture compacting. The compaction started with a cold isostatic pressing at the pressure of 200 MPa. Then, the green compacts were weighed, and the porosity was calculated as between 32–40%. Finally, the green compacts were compacted using a hot vacuum press method at the working temperature interval range from 450 to 470 °C and external pressure of 500 MPa. The density of the samples was determined from weighing and volume measurement in the range from 4.1 to 4.15 g·cm^−3^. The porosity of the finished material was 2.44% ± 0.15%. The microstructure of the experimental material is given in Figure 1, where the black areas are related to the presence of graphite flakes (high carbon content) and the brighter areas are compacted grains of the HDH Ti powder.

### 2.2. Surface Modification Process

**Figure 2:**
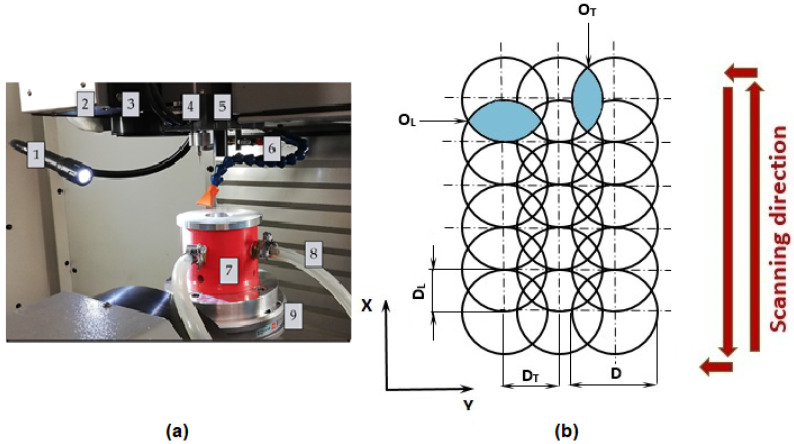
Experimental setup. (**a**) The main parts of the laser equipment; (**b**) scheme of pulse mode. 1—lighting of the workspace, 2—exhaust, 3—laser scanner output, 4—Z-axis measure probe, 5—positioning CCD camera, 6—cooler, 7—fixture for sample placement, 8—inlet of shielding gas, 9—work table; D—laser beam diameter, D_L_—lateral pulse distance (µm), O_L_—lateral pulse overlap (%), D_T_—transverse pulse distance (µm), O_T_—transverse pulse overlap (%).

Within the experiment, the Ti-graphite composite samples were treated by a laser beam in the Ar shielding atmosphere. Before laser beam machining, the sample was cut on a Buehler IsoMet 1000 (Buehler Ltd., Lake Bluff, IL, USA) precision cutter. A weight of 150 g was used, and the blade speed was set to 220 rpm. The cut samples were then ground with P1200 (15.3 µm) Buehler CarbiMet emery paper and then ground with P1200 (15.3 µm) Buehler CarbiMet emery paper. The surfaces were prepared for laser beam ablation after cutting and grinding the samples. Laser ablation of the experimental material was performed on a Lasertec 80 Shape machine (DMG Mori GmbH., München, Germany) equipped with a Yb-doped fiber laser system (Figure 2a). The constant parameters of the experimental procedure are listed in Table 1.

Two different square-shaped surfaces (Surface A and Surface B) with a 5 mm side length were ablated by cross-hatching strategy using different combinations of input process parameters according to Table 2 and Figure 2b. In a cross-hatching strategy, the laser beam passes the machined surface in two directions perpendicular to each other. 

Lateral pulse distance (pulse-to-pulse distance), D_L_, was calculated according to the following formula:

(1) $$D_{L}=\frac{v_{s}}{f}$$

Subsequently, lateral pulse overlap (pulse-to-pulse overlap) O_L_ was expressed as (2):

(2) $$O_{L}=\left(1-\frac{D_{L}}{D}\right)\times 100$$

The summary of incident laser pulses in one place is marked as N. The total energy delivered to the irradiated area of the material in one machined layer (E_T_) was calculated as (3):

E_T_ = E_p_ × N (3)

The sample was placed in the fixture (position 7 in Figure 2a) before machining to allow the use of an Ar shielding atmosphere. 

### 2.3. Surface Characterization

The JEOL JSM 7600F (JEOL Ltd., Tokyo, Japan) high-resolution scanning electron microscope (SEM) was utilized to observe the topography of laser-treated surfaces. The treated surfaces were observed in a secondary electron imaging regime with the following parameters: U = 15 keV, I = 1.0 nA and WD = 15 mm. The ablated surfaces were observed at magnifications from 50 to 1500×.

The elemental composition was measured by an Oxford Instruments Inca X-Max 50 mm^2^ energy-dispersive X-ray spectroscope (Oxford Instruments, Oxford, UK) operated at the same parameters as SEM. The elemental composition of the surface was measured three times for each sample. 

The surface roughness parameters (Ra, Rsk, Rku, RSm and Rmr) were measured using a Mitutoyo SJ 210 (Mitutoyo Europe GmbH, Neuss, Germany) contact-gauge profilometer according to ISO 4288:1997 Standard [46].

The area surface roughness parameters of the ablated surfaces were measured by the ZEISS LSM 700 laser scanning confocal microscope (Carl Zeiss Microscopy GmbH, Jena, Germany) according to ISO 25178 Standard. The microscope is equipped with a 405 nm wavelength laser. The achieved data were processed in ZEN 2009 software and then evaluated in the form of 3D color topography maps using ConfoMap Premium 7.2 software. All surfaces were scanned across the area with the dimensions of 454 × 454 µm^2^ at the magnification of 200× by the EC “Epiplan-Neofluar” 20×/0.50 HD M27 objective (Carl Zeiss Microscopy GmbH, Jena, Germany).

The phase composition of the laser-treated surfaces was studied using a Brucker D8 diffractometer (XRD) (Brucker, Billerica, MA, USA), with the Cu anode (λ = 1.5406 Å). The following parameters were used during the measurements: U = 40 kV and I = 30 mA. The analysis of diffractograms was carried out using Panalytical HighScore Plus software, version 3.0e (Malvern Panalytical B. V., Almelo, The Netherland).

### 2.4. In Vitro Cellular Evaluation

#### 2.4.1. Cell Culture

Human mesenchymal stem cells (hMSCs) were obtained from the bone marrow by aspiration of the bone marrow from the posterior iliac crest after signed informed consent and the approval of the Ethics Committee. Bone marrow mononuclear cells were isolated by centrifugation in Ficoll-Histopaque (Sigma-Aldrich, St. Louis, MO, USA) and initially expanded in α-MEM medium (Life Technologie, Carlsbad, CA, USA) with 10% non-heat inactivated fetal bovine serum (FBS) (Termo Fisher Scientific, Waltham, MA, USA). hMSCs from the initial expansion were once passaged and then stored frozen in liquid nitrogen till their usage. Then the cells were incubated in a standard cultivation medium (α-MEM medium supplemented with 10% heat-inactivated FBS, penicillin (20 U/mL; Sigma-Aldrich, St. Louis, MO, USA) and streptomycin (20 mg/mL; Sigma-Aldrich, St. Louis, MO, USA). In general, cells were cultivated in an incubator at 37 °C and 5% CO_2_ atmosphere. The experiments were performed using hMSCs from healthy donors (*n*= 2) with a passage number from four to five.

#### 2.4.2. Metabolic Activity Determination

Cells were seeded on the Ti-graphite samples located on the bottom of 24-well plates (TPP, Switzerland) at the concentration of 15,000 cells/cm^2^ in the standard cultivation medium for 24 h. After this period, the samples with adhered cells were transferred to a new 24-well plate and incubated for an additional 48 h. Then, the medium was aspirated, and a staining solution for metabolic activity determination was added. A metabolic activity test (Cell Titer 96 AQueous One Solution Cell Proliferation Assay, MTS, Promega, Madison, WI, USA) was performed according to the standard protocol of the manufacturer (the reduction of MTS reagent to a colored formazan product was induced by metabolically active cells). The supernatants from the samples were transferred into 96-well plates, and their absorbance was measured using a multi-detection microplate reader (Spark, Tecan, Switzerland) at 490 nm and reference at 655 nm. The measured results were normalized to the size of the sample and expressed relative to the control cells (set as 100%) cultivated on the standard tissue culture-treated polystyrene (CTRL-PS) as a percentage.

#### 2.4.3. Osteogenic Differentiation of hMSCs

After the determination of their metabolic activity, the hMSCs were washed in PBS (phosphate saline buffer), and the osteo-differentiation medium was added to them for 14 days (standard α-MEM medium (Life Technologies, USA) with 10% heat non-inactivated FBS (PAA, Austria), penicillin (20 U/mL; Sigma-Aldrich, USA) and streptomycin (20 mg/mL; Sigma-Aldrich, USA) supplemented with 0.5 mM sodium L-ascorbate, 10 mM glycerol-phosphate and 0.1 μM dexamethasone, changed every 3 days for fresh supplemented medium). Then, the cells were fluorescently stained using a specific antibody against osteocalcin protein and visualized by fluorescence microscopy.

#### 2.4.4. Fluorescence Staining of Cells and Microscopy

The cells on Ti-C samples were fixed by 4% paraformaldehyde in PBS at room temperature (RT) for 15 min, and permeabilized by 0.1% Triton X-100 in PBS (Sigma-Aldrich, USA) at RT for 20 min. For morphology detection, actin filaments were stained with Alexa Fluor 488 Phalloidin (ThermoFisher Scientific, USA) for 45 min at 37 °C, and nuclei with DAPI (Sigma-Aldrich, USA) for 15 min at 37 °C. For osteo-differentiation detection, after fixation and permeabilization, the samples were incubated with primary polyclonal antibody against osteocalcin (Abcam, UK) (1 h at 37 °C), then with secondary fluorescently tagged antibody (AlexaFluor555 goat anti-rabbit—Invitrogen, USA (1 h at 37 °C)). Images of the cells were acquired using the Olympus IX71 microscope (Olympus, Hamburg, Germany) equipped with a color-cooled camera DP74.

### 2.5. Statistical Analysis

Statistical evaluation of the evaluated parameters was performed using Minitab v. 17 software (Minitab, LLC, State College, PA, USA). The data were tested for normal distribution, and a one-way ANOVA was applied. For the statistical tests, the levels of significance were set at 95% (α = 0.05) and 99% (α = 0.01).

## 3. Results and Discussion

### 3.1. Scanning Electron Microscopy (SEM) Surface Observation

**Figure 3:**
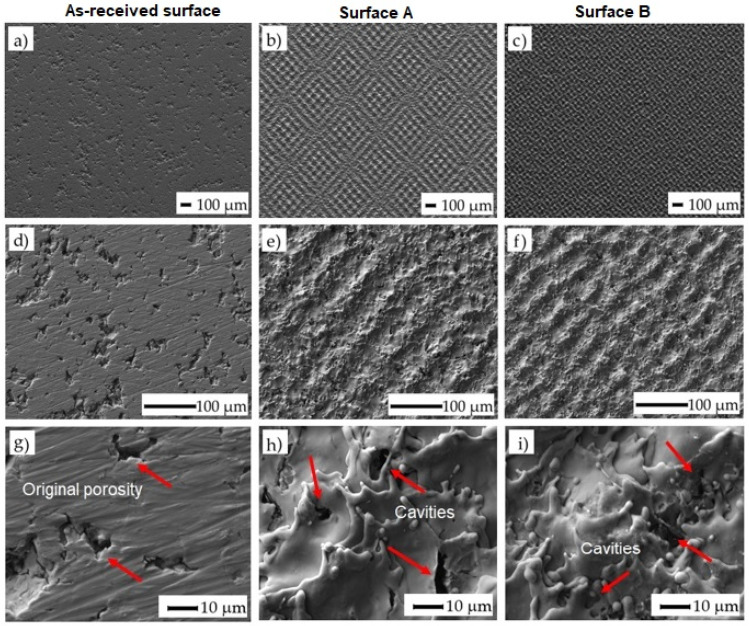
SEM images of treated surfaces: (**a**) as-received surface at a magnification of 50×; (**b**) surface A at ET of 0.5 mJ and at a magnification of 50×; (**c**) surface B at ET of 5 mJ and at a magnification of 50×; (**d**) surface before laser machining at a magnification of 250×; (**e**) surface A at ET of 0.5 mJ and at a magnification of 250×; (**f**) surface B at ET of 5 mJ and at a magnification of 250×; (**g**) surface before laser machining at a magnification of 1500×; (**h**) surface A at ET of 0.5 mJ and at a magnification of 1500×; (**i**) surface B at ET of 5 mJ and at a magnification of 1500×. The arrows indicate the location where the cavities were formed.

The surface morphologies of the as-received surface and the surfaces after laser machining are shown in (Figure 3). Variously broken wavy formations of the remelted and solidified material were observed on both machined surfaces. The texture of the surface machined by applying a higher amount of energy transmitted to the material (E_T_ = 5 mJ) (Surface B) appears to be slightly finer.

The square-shaped texture resulting from the used cross-hatching strategy of laser beam movement can be observed when the incident energy is higher. At higher magnification (1500×), the cavities at the sizes ranging between 1 and 5 µm were detected in both machined surfaces. These micro defects probably resulted from the interaction of the melted material with the shielding gas flow. Such porosities are generally caused by entrapped gases in the molten material owing to the high laser energy or unstable process conditions. In our case, the final shape and dimension of the porosity resulted from the original porosity of the machined material prepared by the powder metallurgy and the porosity induced by the laser irradiation [47]. Using this combination, it is possible to manufacture the surfaces and subsurface layers with interconnected pores, thereby promoting cell growth in these areas.

### 3.2. Energy-Dispersive X-ray Spectrometry (EDS) Analysis

**Figure 4:**
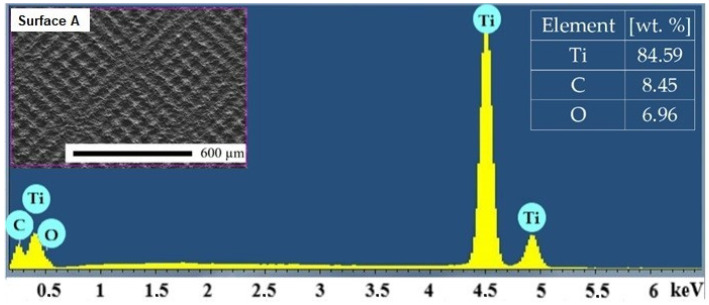
Representative EDS spectrum of Surface A.

The mean values and standard deviations of the non-machined and machined surfaces are given in Table 3. The representative EDS spectrum obtained from one point on Surface A is shown in Figure 4. Titanium (Ti), carbon (C) and oxygen (O) were detected in all the analyzed surfaces. It is noticeable that the minimal average weight percentage of oxygen was observed on the as-received surface. The content of the oxygen increased with the increasing amount of total energy transmitted to the material [48].

### 3.3. Surface Roughness Measurement Evaluation

**Figure 5:**
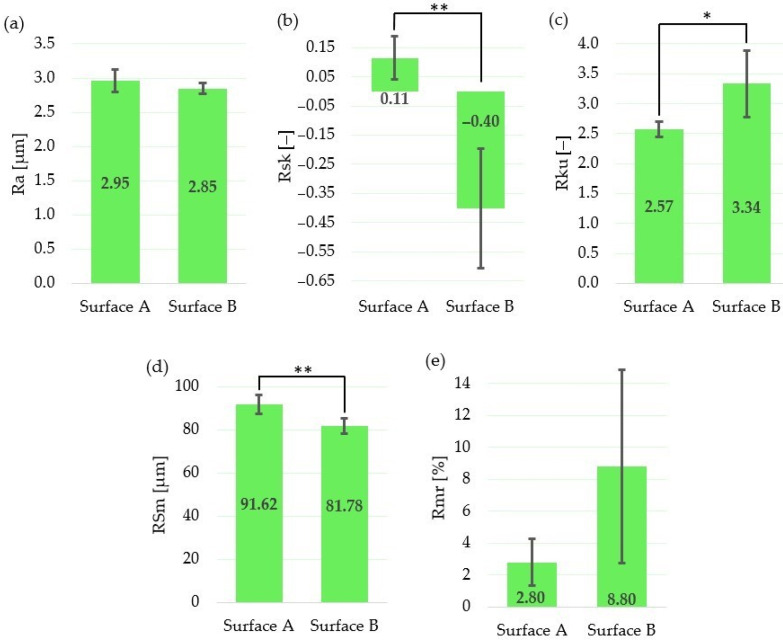
Evaluation of surface roughness parameters for Surface A and Surface B: (**a**) arithmetical mean height Ra; (**b**) mean width of the profile elements RSm; (**c**) relative load length ratio Rmr; (**d**) skewness Rsk; (**e**) kurtosis Rku; * statistically significant difference between means of roughness parameters at the significance level of 0.05; ** statistically significant difference between means of roughness parameters at the significance level of 0.01.

**Figure 6:**
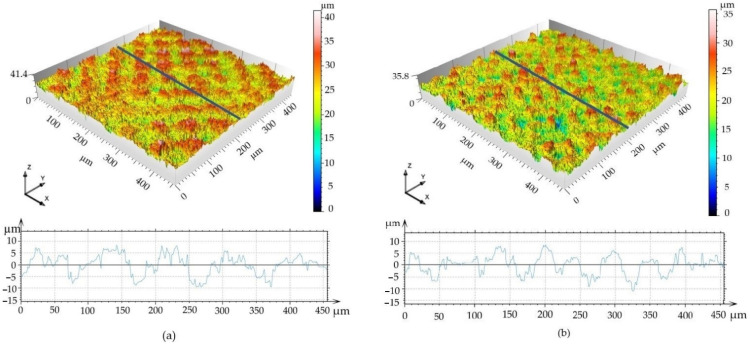
3D surface maps. (**a**) Surface A; (**b**) Surface B.

The mean and standard deviation values of the average arithmetic value of roughness (Ra), skewness (Rsk), kurtosis (Rku), mean width of the profile elements (RSm) and material ratio of the surface profile (Rmr) with one-way ANOVA statistics are depicted in Table 4 and Table 5 and Figure 5. Color 3D maps of the machined surfaces with profiles in the *x*-axis direction are shown in Figure 6.

The results show that, although the amplitude parameter (Ra) and the parameter which expresses the bearing length ratio (Rmr) are not statistically different, the skewness (Rsk) and spacing parameter (RSm) are statistically different at the statistical level of 0.01. The statistically significant difference between means at the significance level of 0.05 was observed for the kurtosis (Rku).

The mean value of the skewness for Surface A is positive; on the other hand, it is negative for Surface B. That means that neither Surface A nor Surface B exhibit symmetrical height distribution. It can be concluded that the surface with positive Rsk (Surface A) is more porous, while the surface with negative Rsk (Surface B) is rougher. These results correspond with the findings of Tavakoli et al. [49], who observed positive Rsk for the Ti6Al4V laser irradiated surfaces with low energies; however, when using higher laser energies, the Rsk was negative.

According to the results of the roughness measurement, the distribution curve of the Surface A profile is platykurtic (Rku <3; the profile consists of relatively few flat peaks and valleys), while the distribution curve of Surface B profile is leptokurtic (Rku >3; the profile consists of relatively many sharp peaks and valleys).

Finally, the difference between the mean values of the profile peaks spacing values (RSm) is approximately 10 µm, with a higher value in the case of Surface A.

Although we did not find significant differences between roughness parameters, Ra, Rmr, there are significant differences between surface profiles parameters, Rsk, RSm and Rku, that have higher relevance at a cellular level in the osseointegration process. The more preferable profiles with flat and large interfacial areas [50] were obtained by applying a lower level of the transferred energy, controlled by applying low output laser power, lower pulse frequency and higher scanning speed.

Not only the surface roughness (Ra, Sa) impacts the adhesion and proliferation of mesenchymal stem cells, Sdr and Sku should also be used as evaluation indicators. Especially as stem cells have a higher rate of proliferation on the flat and large interfacial area.

The 3D surface maps indicate that the areas above the mean line in Surface A are double the size of those in Surface B (785 versus 345 μm^2^, respectively); and, similarly, the areas under the mean line are larger for Surface A compared to those for Surface B (922 versus 403 μm^2^, respectively).

### 3.4. X-ray Diffraction (XRD) Observation Results

**Figure 7:**
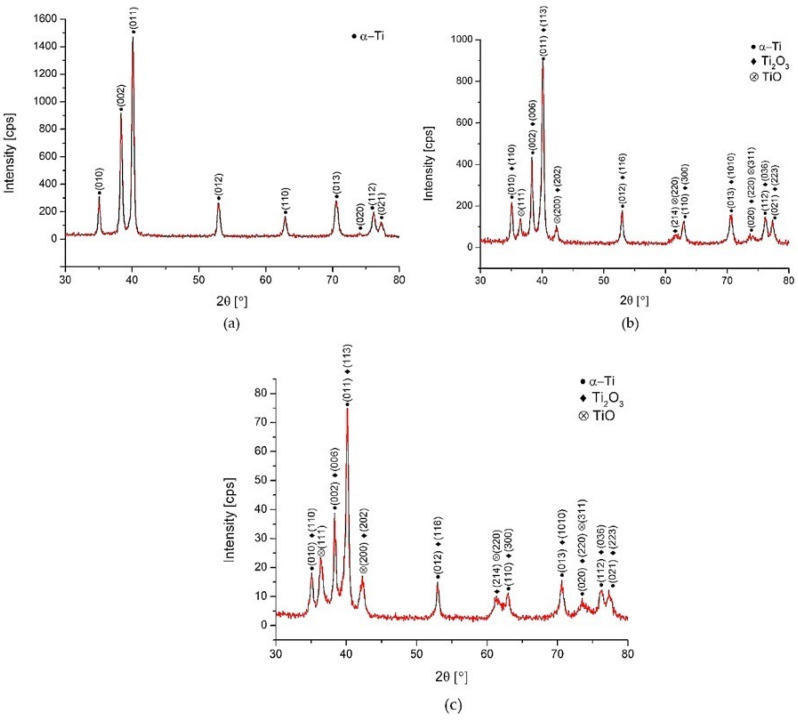
XRD patterns. (**a**) as-received surface, (**b**) Surface A, (**c**) Surface B.

The XRD patterns of Surface A, Surface B and the as-received surface are depicted in Figure 7. They confirm the formation of two types of oxides (TiO and Ti_2_O_3_) on the laser machined surfaces. There was no XRD peak confirmed at around 25.3°, which could correspond to the thermodynamically most-stable TiO_2_ (rutil or anatase). The much lower intensity of oxide peaks of the Surface B treated at higher energy (E_T_ = 5 mJ) can be related to lower thicknesses of the grown oxidic films. Contrary to the results of EDS evaluation where the presence of carbon up to ~9.5 wt. % was observed, no diffraction peaks corresponding to carbon were detected. This suggests that carbon could be present on the surface of laser-treated samples in the form of amorphous graphite undetectable by XRD observation.

The results correlate with the findings in [48,51], where the authors claim that the top layer of the film produced on titanium was not a pure TiO_2_, and it also can be supposed that TiO_2_ was probably contained in the amorphous structure that is formed due to the extremely fast heating/cooling rates and non-isothermal features, and therefore, no diffraction peaks were detected. The difference between the XRD patterns of Surface A and B were insignificant.

These experimental XRD patterns match well with the International Centre for Diffraction Data (ICDD) reference cards n° 03-065-9622 (titanium), n° 00-002-1196 (TiO) and n° 01-071-1047 (Ti_2_O_3_).

### 3.5. Biocompatibility and Osteoinductivity Evaluation

**Figure 8:**
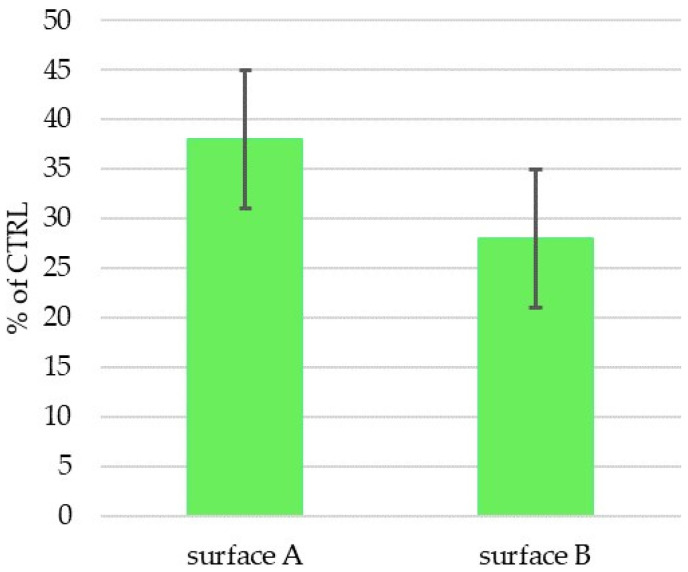
Metabolic activity/cell number of hMSCs cultivated for 72 h on Ti-graphite samples with Surface A and Surface B (activity/cm^2^ related in percentage to cell activity on control sample (CTRL-PS)). No statistically significant difference between Surface A and Surface B was observed at the significance level of 0.05.

**Figure 9:**
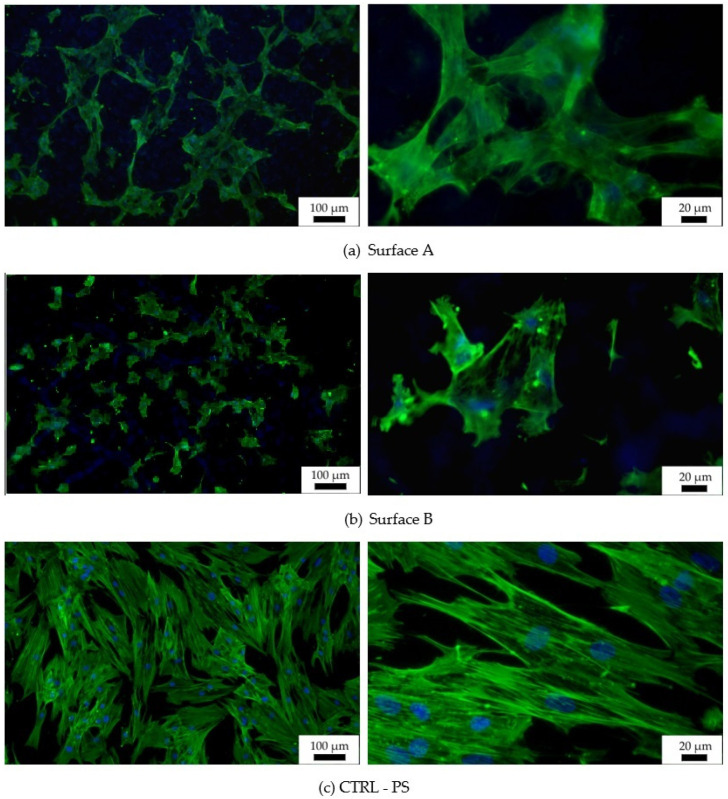
Fluorescent images of hMSCs cultivated for 72 h on graphite-Ti samples. Staining of actin by PhalloidinAlexaFluor 488 (green) and of the nucleus by DAPI (blue); (**a**) Surface A, (**b**) Surface B, (**c**) surface of the control tissue-culture treated PS sample (CTRL—PS).

The two prepared and characterized laser machine surfaces A and B were tested in vitro using human mesenchymal stem cells (hMSCs) to determine their biocompatibility and osteoinductivity. hMSCs were cultivated on the experimental surfaces, as well as on a control tissue-culture-treated plastic (CTRL-PS), and their metabolic activity/cell number was determined after 72 h of incubation (Figure 8). The cells on Surface A grew more than those on Surface B in individual experiments (cell batches); however, the summarized data did not reveal any significance due to the high standard deviation. In comparison to the control plastics, metabolically produced color reaction by active cells on both surfaces tested significantly decreased to 40% of the control one, which may indicate the reduced cell number and/or metabolism on the prepared surfaces. The reduced metabolism of hMSC on structured surfaces versus flat ones was already described [52]; however, in our case, it is most probably related to the reduced initial cell adhesion to the surface rather than to the decreased cell metabolism, as the cell morphology (Figure 9) corresponds with the alive and proliferating cells.

Images from fluorescence microscopy (Figure 9) support this result, showing that the number of cells on both tested surfaces was smaller than the number of cells on the control plastic. Moreover, the hMSCs morphology of individually tested surfaces, as well as that of the reference surface, differed. The most widely spread cells appeared on the flat plastic surface, which was specially treated for tissue-culture cultivation by the manufacturer, while the cells on the machined surface were spread just according to their specific profile. More widely spread and mutually interconnected cells grew on Surface A, which is characterized as the surface with “wide valleys” (Figure 6a) (valley area is comparable to the cell size [53]). On the other hand, smaller and isolated cells could be found on Surface B, where rather sharp peaks alternated with relatively narrow valleys that were smaller than the regular cell size. Thus, the particular material has a slight influence on the initial cell growth while causing an apparent change in the cell morphology. The cells tried to fit into the provided space; thus, their behavior was directly related to it. Many existing studies show that different surface properties attract different cell types, thus tuning the material surface to the particular desired cell type can be attracted to it, and, on the other hand, can work as a repellent for other cell types [16,17].

**Figure 10:**
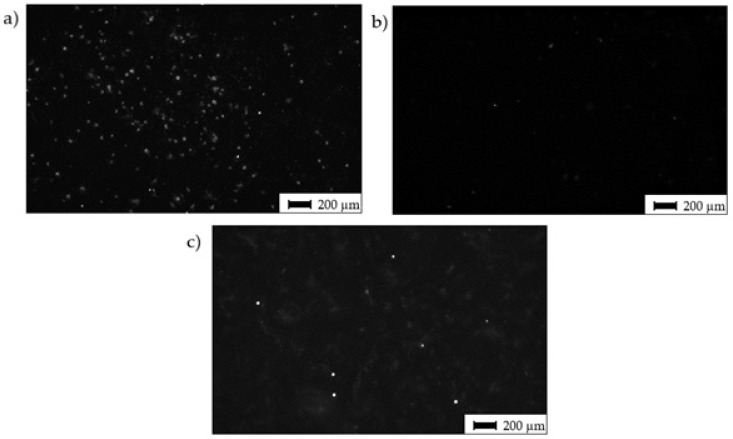
Osteo-differentiation of hMSCs—black and white images of hMSCs cultivated for 14 days in osteo-differentiation medium. Staining of antibody against osteo-marker osteocalcin; (**a**) Surface A; (**b**) Surface B; (**c**) surface of the control sample (CTRL—PS).

Interestingly, despite alike behavior after 72 h of incubation, the cells cultivated for 14 days in an osteo-differentiating medium on the different surfaces revealed different osteoinductive/conductive abilities (Figure 10). The highest fluorescence signal from the detection of osteogenic marker osteocalcin was documented in the cells cultivated on Surface A. This signal is even higher than the signal on the control plastics, which indicates enhanced osteoinductive properties of Surface A. The graphs in Figure 6a indicate that the curve (profile) of Surface A is more ragged than that of Surface B, thus providing not only micro-topography but also submicro- or nano-topography. The more structured surface can thus better mimic the original bone structure, thereby positively influencing the osteoinduction of hMSCs, as has been previously reported many times [54,55].

It is well known that surfaces with microscale irregularities along with surface chemistry promote bone cell attachment, new bone integration and adhesion between the bone tissue and implant [56], but a more accurate quantitative characterization is still not entirely sufficient. This study, following previous studies of the authors, enhances the knowledge on the significance of the surface micro profile parameters Rsk, Rku and RSm, through which cell attachment and proliferation can be modulated. As modern trends in the research of biomedical materials surface modification prefer the need to develop surfaces that not only improve the bio-activeness but also improve the antibacterial effect [57], the quantification of surface profile parameters on the micrometer and nanometer-scale is necessary for providing effective results.

Each surface has specific properties that can affect cells in different ways. Although the results of this study revealed there was a difference in the specific surface properties that led to the cell behavior in a different way, one important limitation needs to be considered. The cell’s activity on the treated surface is a result of a complex interaction of many variables, and not all of them could be evaluated within this study; therefore, further research is needed on the reason for these results. The influence of the surface profile, together with the surface chemistry and surface energy on the hMSCs growth and osteo-differentiation, need to be observed in further research.

## 4. Conclusions

In this study, the surfaces of the powder metallurgy-processed Ti-graphite composite were machined by applying different laser beam energies delivered to the material in the same location, and the surface integrity parameters were studied concerning the in vitro bioactivity. Based on the conducted experiments, the following considerations can be drawn:

(1) The obtained results confirmed that the energy directly influences the surface chemistry, morphology and roughness parameters, which determine their biocompatibility and osteoinductivity.
(2) It was indicated that the introduction of a lower energy amount (ET = 0.5 mJ) into the workpiece material resulted in a surface profile with a few wide and high peaks with rugged surface and a few low and wide valleys and higher peaks—Surface A.
(3) The profile that consists of relatively a lot of narrow (sharp) peaks and low valleys, with the valleys dominating over the peaks, was documented when a higher level of energy was used (ET = 5 mJ)—Surface B.
(4) The in vitro analysis using hMSCs revealed that the surface produced by applying the lower level of incident energy promotes cells’ growth and osteo-differentiation, when compared with the surface machined using higher energy level.
(5) It was confirmed that skewness, kurtosis and width of the surface profile elements are important variables influencing hMSCs growth and osteo-differentiation.
(6) The adhesion and proliferation behavior of cells on the surface is the result of a complex interaction of many variables, and therefore, the surface energy of the laser-modified surfaces, in relation to surface profiles and chemistry, will be investigated in more detail in further studies.

## Figures and Tables

**Table 1:** Constant parameters of the experimental setup.

Parameter	Value
Wavelength of laser radiation λ	1064 nm
Pulse duration τ	120 ns
Spot diameter D	50 µm
Transvers pulse distance (line-to-line distance) D_T_	10 µm
Transvers pulse overlap (line-to-line overlap) O_T_	80%
Ablated layers	2
Argon flow rate	10 L·min^−1^

**Table 2:** Parameters of laser treatment.

Surface	Output Power (W)	f (Hz)	v_s_ (mm·s^−1^)	D_L_ (µm)	O_L_ (%)	N (-)	E_T_ (mJ)
A	4	20	2000	100	no overlapping	2.5	0.5
B	20	100	1000	10	80	25	5

**Table 3:** Elemental composition of the as-received surface, Surface A and Surface B.

Element (wt. %)	As-Received Surface	Surface A	Surface B
Ti	86.35 ± 0.06	84.32 ± 0.56	75.18 ± 0.56
C	8.15 ± 0.07	8.58 ± 0.22	9.52 ± 0.28
O	5.51 ± 0.02	7.10 ± 0.35	15.30 ± 0.30

**Table 4:** Surface roughness parameters measurements results.

Roughness Parameter	Surface A	Surface B
Ra (µm)	2.95 ± 0.16	2.85 ± 0.08
Rsk (-)	0.11 ± 0.08	−0.40 ± 0.21
Rku (-)	2.57 ± 0.13	3.34 ± 0.56
RSm (µm)	91.62 ± 4.39	81.78 ± 3.61
Rmr (%)	2.80 ± 1.45	8.80 ± 6.07

**Table 5:** One-way ANOVA (Fisher’s) comparison of measured parameters of Surface A and B.

Roughness Parameter	F-Value	*p*-Value	R^2^	Pooled SD
Ra	1.36	0.277	14.54	0.143
Rsk	22.17	0.002 **	73.48	0.173
Rku	7.30	0.049 *	41.19	-
RSm	12.00	0.009 **	60.01	4.49
Rmr	3.70	0.120	23.6	-

Note: * *p* < 0.05, ** *p* < 0.01.

## Data Availability

The data presented in this study are available on request from the corresponding author.
